# Crisis management in Korean Generation Z’s leadership expectations: a robustness analysis

**DOI:** 10.3389/fpsyg.2026.1925743

**Published:** 2026-08-19

**Authors:** SeBi Lee, Jin Hee Choi, Guy Major Ngayo Fotso

**Affiliations:** 1 aSSIST University (Seoul School of Integrated Sciences and Technologies), Seoul, Republic of Korea; 2 Franklin University Switzerland, Sorengo, Switzerland; 3 Seoul Business School, aSSIST University, Seoul, Republic of Korea; 4 HEG School of Management Fribourg, University of Applied Sciences and Arts of Western Switzerland, Fribourg, Switzerland

**Keywords:** crisis management, Generation Z, ipsative analysis, leadership expectations, leadership perception, response style, social role theory, South Korea

## Abstract

Generational expectations of leaders are typically inferred from competency importance ratings, but such ratings are difficult to interpret when they are subject to strong ceiling effects. We examine this methodological challenge in South Korean Generation Z, using crisis management as a focal test case. Drawing on a June 2024 nationwide quota sample of 1,000 South Koreans aged 14–28, we measured the importance respondents assigned to eighteen twenty-first-century leadership competencies and analyzed the ratings with paired comparisons, bootstrap resampling, and within-person centering. Crisis management received the highest mean rating, and its first place was highly stable under resampling, yet strong ceiling compression left it statistically indistinguishable from communication skills; its top position therefore indicates high salience within broadly shared expectations, not a singular priority. Women reported higher raw ratings for crisis management, but the difference was no longer significant after within-person centering, and an equivalence test rejected effects as large as the conventional small-effect benchmark, a pattern consistent with a difference in scale use rather than in priorities. Women’s stronger emphasis on social skills remained robust after the same correction, in line with social role theory. The cross-sectional design supports neither causal nor generational claims. What the study does establish is twofold: top-ranked means and raw demographic gaps in compressed ratings cannot be interpreted without corrections for ceiling effects and scale use, and crisis management is robustly salient in a cohort where prior research had placed it at the periphery.

## 1 Introduction

Crisis management has moved from a specialized organizational function toward a broadly held expectation of contemporary leaders. As organizations face recurring disruption, the capacity to make sense of acute threat and to act decisively under uncertainty now figures prominently in what people expect of leaders (Bundy et al., 2017; Pearson and Clair, 1998). For the generation now entering work, a basic question follows: which of the many competencies attributed to twenty-first-century leaders do they rate as most important, and why? The question concerns leadership perception, the qualities people expect of leaders rather than the behavior leaders enact, and about how survey ratings capture such expectations.

The question is especially pointed in South Korea. Korean Generation Z has come of age within a structurally volatile setting: chronic security tension, external economic exposure, and intensifying competition over strategic industries (Akoto, 2024; Hemmert, 2020), conditions detailed in Section 2.3. Cohort accounts hold that formative-period conditions leave a durable imprint on a generation’s values (Lyons and Kuron, 2014; Parry and Urwin, 2011; Twenge et al., 2010). On this view, sensitivity to disruption may be a broadly shared feature of Generation Z, which makes South Korea, as an unusually concentrated case of such exposure, a strong setting in which to examine how a global generational tendency takes shape under acute national conditions.

Prior work has interpreted individual-level attitudinal patterns in light of broader institutional and economic conditions (Inglehart and Welzel, 2005; Perry and Wise, 1990; Twenge et al., 2010). In that spirit, we treat the salience of crisis management among Korean Generation Z as a pattern that may be read cautiously against the cohort’s broader social and economic context, without claiming that this context produces those preferences.

Historically, crisis management has been conceptualized as a specialized organizational function, typically reserved for executives navigating low-probability, high-consequence events (Pearson and Clair, 1998). Although recent work treats it as a more general leadership expectation as disruption becomes routine (Bundy et al., 2017), it remains framed within organizational management, with leaders responding to threats faced by their organizations. The framework used here originally found crisis management to be peripheral in the millennial leadership literature and salient mainly in accounts of older generations (Ngayo Fotso, 2021). That a competency previously peripheral within the same framework ranks first in this cohort is unexpected and calls for examination.

We therefore consider a modest interpretive possibility: for Korean Generation Z respondents who have come of age amid chronic security tension and structural economic precarity, crisis competence may be regarded less as a specialized, reactive skill than as a basic, broadly held leadership expectation. We do not test this reading directly. We treat the breadth and stability of the priority as consistent with it and develop the interpretation in the discussion.

This study tests how robust the apparent priority of crisis management is for Korean Generation Z. Claims that a generation rates a particular competency most important are common in practitioner and academic discourse, yet they are rarely examined for whether the lead is statistically meaningful, stable across groups, or free of response-style artifacts. Drawing on importance ratings from 1,000 respondents, we ask three questions: (RQ1) where crisis management ranks in mean importance, and whether its lead over the next competency is statistically meaningful; (RQ2) whether that rank is stable across academic field, region, age, occupational status, and gender, and under resampling; and (RQ3) whether the higher ratings women give crisis management reflect a different priority or a different use of the scale. The discussion additionally offers an interpretive, untested reading of the observed pattern against Korea’s broader social and economic context. The present paper is confined to the salience and stability of a single competency. As an exploratory secondary analysis, it reports observed patterns in a population underrepresented in generational leadership research. We use “Generation Z” throughout as a label for the sampled age cohort rather than as a claim about a distinct generational effect, since a single-wave design confounds cohort with age and period (Rudolph et al., 2018). The study also speaks to a question of interpretation for leadership perception research: how to read a top-ranked competency when ratings are compressed, since a competency can rise to the top of what a cohort expects of leaders without being singled out above the next-ranked qualities.

## 2 Theoretical background

### 2.1 Crisis management as a core twenty-first-century leadership competency

Crisis management denotes the leadership work of anticipating, containing, and recovering from acute, high-uncertainty disruption. Early work framed crisis response as a process of enacted sense-making under conditions of equivocality (Weick, 1988). Pearson and Clair (1998) recast crisis management as an organizational capability spanning prevention, response, and recovery rather than a reactive afterthought, and integrative reviews since then treat crises as low-probability, high-consequence events demanding sense-making, decisive action, and credible communication (Bundy et al., 2017). Research on crisis leadership shows that competent performance rests on a phase-specific repertoire (signal detection, prevention, containment, recovery, and learning) rather than on a single trait (James and Wooten, 2005; Wooten and James, 2008), and reviews converge on a compact competency set of sense-making, communication, adaptability, decisiveness, and care (Boin et al., 2016). A related strand extends this work to extreme contexts, where physical or psychological danger raises the stakes of competent leadership (Hannah et al., 2009). Crisis competence has thus become a general, learnable leadership expectation, and a growing research and training agenda treats it as a capacity that can be deliberately developed at local and non-executive levels of responsibility (Eid et al., 2023). Empirical studies likewise show that leaders’ actions during acute crises have measurable effects on employees (Gray et al., 2023), and evidence from the COVID-19 pandemic indicates that relationship-oriented leader behaviors gain particular weight under crisis conditions (Sedefoglu et al., 2024). Sector-specific studies similarly examine crisis management as a skill of managers themselves, for example among sport managers (Atılgan and Kaplan, 2022). Even so, this expanded view still locates the competence in an organizational leader responding to an organizational threat, and it says little about whether ordinary individuals, rather than executives, rate it as broadly important.

### 2.2 Generation Z and crisis-related leadership expectations

Generational cohort theory holds that formative-period conditions shape enduring values (Parry and Urwin, 2011), though the field cautions that within-cohort variation often rivals between-cohort variation and that single-wave data cannot isolate cohort effects (Costanza et al., 2012; Lyons and Kuron, 2014; Ravid et al., 2024). A critical review goes further, holding that single-time-point designs perfectly confound cohort with age and period and urging caution before workplace patterns are attributed to generational membership (Rudolph et al., 2018). The present study adopts this caution and treats its findings as a description of one cohort’s current competency priorities rather than as evidence of a generational effect. Generation Z matured through a period unusually dense with disruption, and comparative work documents distinctive cohort expectations (Anderson et al., 2017; Egerová et al., 2021; Twenge et al., 2010). These accounts motivate the expectation that crisis competence is salient for this cohort, an expectation the present study examines by testing it as a top-ranked, broadly shared priority. Practitioner-facing accounts likewise describe a cohort entering work with heightened expectations shaped by economic and social uncertainty (Schroth, 2019). Generational identity is itself multidimensional, not uniform (Costanza and Finkelstein, 2015), and studies of Generation Z employees report strong responsiveness to leadership and to opportunities for voice (Katsaros, 2025). In Korea specifically, generational differences in work values have been documented among government employees (Park and Park, 2024).

How this expectation compares across national cohorts is not yet well charted. Western accounts of Generation Z at work emphasize meaning, flexibility, voice, and developmental support (Anderson et al., 2017; Schroth, 2019), while comparative European evidence stresses security and stable employment alongside these expectations (Egerová et al., 2021). An integrative review of the same eighteen-competency framework found that crisis management was not emphasized in the millennial leadership literature and figured mainly in accounts of older generations (Ngayo Fotso, 2024a). Seen against prior accounts, a first-place standing for crisis management in a Korean Generation Z sample is comparatively distinctive, which is why the present study examines how far it is shared and how it should be interpreted, rather than assuming either.

### 2.3 The Korean political-economic and geopolitical context

Because the qualities people come to expect of leaders are shaped partly by the conditions they live through, the national setting matters here. South Korea offers a concentrated setting of crisis-related exposure. The peninsula remains divided and subject to recurring security tension, the trade-dependent economy is sensitive to external shocks, and the country sits at the front line of competition over strategic industries such as semiconductors and batteries (Akoto, 2024; Hemmert, 2020). For young people this coincides with entrenched precarity: persistently high youth unemployment and NEET rates (OECD, 2019), a growing concentration in insecure work (Lee and Baek, 2025; Lee and Green, 2025), some 414,000 people aged 15–29 withdrawn from the labor market in 2023 (Statistics Korea, 2024b), the world’s lowest total fertility rate (0.72 in 2023; Statistics Korea, 2024a), and acute housing unaffordability, including jeonse-deposit fraud with more than 30,000 recognized victims concentrated among people in their twenties and thirties (Ministry of Land, Infrastructure and Transport, 2023, 2025). Such exposure is experienced at the level of society rather than the individual discipline, region, or workplace. We measure none of these conditions at the individual level, however, so the macro-environment is offered as interpretive background, not as a variable linked to the ratings.

### 2.4 Perceived insecurity and leadership priorities

Because the study is exploratory, we treat theory as an interpretive lens rather than a hypothesis to test. The reading developed below draws on two ideas: that sustained societal insecurity can reshape individual priorities (Inglehart and Welzel, 2005), and that, under conservation-of-resources logic, resource loss looms larger than resource gain and losses cascade in spirals, so that sustained threat leads people to safeguard valued resources and to prioritize stability and security (Hobfoll, 1989; Sverke et al., 2002). Consistent with this, scarcity research shows that deprivation pulls attention toward immediate threats (Mani et al., 2013), a preference for security strengthens during the transition to adulthood (Rouvroye et al., 2024), and existential insecurity during the COVID-19 pandemic measurably shifted societal values in Japan (Akaliyski et al., 2024). The circumstances of many young Koreans fit the loss-spiral description: insecure employment, unaffordable housing, and foregone family formation compound one another. Where scarcity takes this form, the theory predicts that people come to prize the capacity to arrest and contain disruption and, applied to leadership, that they weigh crisis competence heavily among the qualities they expect of leaders. Our data are consistent with this prediction, though a single-country design can neither confirm nor falsify the reading, since almost any ranking could be reconciled with the same context after the fact. The reading acquires evidential value only under a comparison that includes a lower-risk cohort, which we identify as the decisive test of the contextual account.

### 2.5 Who might value crisis competence most?

If the priority reflects a society-wide experience, it should be broadly shared, yet several factors could still shift emphasis. Disciplinary socialization could lead STEM and non-STEM students to weigh competencies differently (Biglan, 1973); regional and developmental context could matter across the cohort’s wide age span; and social role theory predicts gendered differences, with women weighing relational and communal competencies more heavily (Badura et al., 2018; Eagly and Wood, 2012; Koenig et al., 2011; Paustian-Underdahl et al., 2014). Testing crisis-management importance against each partition shows how far it is a shared signature and where, if anywhere, it varies.

### 2.6 Measuring leadership-competency priorities

To rank priorities, the study uses the eighteen-competency inventory that Ngayo Fotso (2021) derived from a review of the leadership literature, a theory-neutral list spanning adaptability, cognitive and communicative skills, global and collaborative leadership, digital competence, sustainability, and crisis management. The inventory has since been examined across generations (Ngayo Fotso, 2024a) and national settings (Ngayo Fotso, 2024b). The inventory provides a common metric on which the relative importance of crisis management can be assessed. The present study uses mean importance to locate crisis management in the ranking and does not model the dimensional structure of the inventory.

## 3 Materials and methods

### 3.1 Data and participants

The data are importance ratings from 1,000 Korean Generation Z respondents aged 14–28 at the time of the June 2024 survey, evenly split by gender (500 men, 500 women). The age range corresponds to birth years of approximately 1996–2010, a range used for Generation Z in recent peer-reviewed work with the same 14–28 age span (Piepiora et al., 2024) and consistent with the most frequently cited definition of about 1995–2010 (Benítez-Márquez et al., 2022); it is close to the widely used cutoff of 1997 onward (Pew Research Center, 2019). The oldest respondents sit at the Millennial and Generation Z boundary, and the bounds were set by survey eligibility rather than by a narrower generational claim. Macromill Embrain, a professional research-panel provider, administered the survey online using quota sampling, with interlocking quotas for gender, three age bands (14–19, 20–23, 24–28), and the seventeen first-level administrative regions set to approximate their population distribution; respondents were drawn from the provider’s standing opt-in panel. Respondents spanned all major academic fields, the seventeen regions, and a range of occupational statuses. A sample of 1,000 matches the scale of comparable panel-based cohort studies and yields high precision for the present descriptive aims: each competency mean is estimated with a 95% confidence interval of roughly ±0.07 scale points, and the gender and subgroup contrasts retain adequate sensitivity to detect small effects (*d* ≈ 0.18–0.20). These figures are *post hoc* precision statements, not an *a priori* power analysis; the sample size was set by the survey design, not by a power calculation. For the gender contrasts specifically, this sensitivity bound means that effects smaller than *d* ≈ 0.18 could not be reliably detected, a constraint addressed with an equivalence test in Sections 3.3 and 4.4. The study reanalyzes fully anonymized data and is exploratory. The analyses reported here were not preregistered; because the study is a secondary analysis of an existing dataset, it poses research questions instead of hypotheses, and the directional expectations in Section 2.5 serve only to organize the subgroup analyses.

### 3.2 Measures

The perceived importance of each leadership competency was measured with the eighteen-item inventory of Ngayo Fotso (2021), each item rated on a seven-point scale from 1 (not at all important) to 7 (extremely important). The inventory was administered in Korean. The eighteen competency items were translated and culturally adapted from the original English framework (Ngayo Fotso, 2021) in collaboration with the professional survey provider (Macromill Embrain) and pretested for comprehension before fielding. Because each of the eighteen competencies was captured by a single item, the inventory measures eighteen distinct constructs rather than multiple indicators of one latent factor. A confirmatory factor model in the conventional sense is therefore not identified for these data. We accordingly report Cronbach’s *α* not as the reliability of a single scale but as an index of overall inter-item homogeneity, which was high (*α* = 0.937). A value this high across eighteen distinct competencies is itself consistent with a strong general-positivity tendency, the same tendency the within-person analyses are designed to remove. The dimensional structure of the eighteen items is not modeled here. A companion study examines it directly with factor-analytic methods (Lee et al., 2026). Single-item measures can yield acceptable validity for concrete, narrowly defined constructs (Bergkvist and Rossiter, 2007; Diamantopoulos et al., 2012; Wanous et al., 1997), but they preclude construct-level reliability estimation and assume the abstract labels were understood uniformly across the age range, an assumption we revisit in the limitations. Subgroup variables were academic field and STEM status, region (also grouped as capital area versus non-capital), age group, occupational status, and gender. Academic field and STEM status were recorded for the 737 respondents who were not secondary-school students, who have no declared major. Occupational status grouped respondents as students, employed (office workers, civil servants, and the self-employed), freelance, not employed, or other (a heterogeneous residual category). Because the present analysis treats each competency as a distinct rated object, not as an indicator of a latent factor, its conclusions about the salience and rank stability of crisis management do not depend on the factor solution reported there. The high inter-item homogeneity we report (*α* = 0.937, mean inter-item *r* ≈ 0.45) is itself consistent with a strong general evaluative factor. Such a factor could account for the compression, and although it could not by itself produce a first-place position, it could make small differences in item loadings appear as a ranked gap; separating genuine item-specific variance from unequal loading is precisely what the factor-analytic companion study addresses. Our claims are accordingly confined to the salience and rank stability of crisis management, not to its separation from a latent structure.

### 3.3 Analytic strategy

Descriptive statistics ranked the eighteen competencies and located crisis management. The rank and mean of crisis management were then computed within each subgroup, and the concordance of the full ordering across academic fields was quantified with Kendall’s W. Subgroup differences in overall importance were tested with *t*-tests and one-way ANOVA, and gender differences item by item with Bonferroni correction (*α* = 0.05/18 = 0.0028); the family of five subgroup mean-level tests (field, region as capital versus non-capital and across the seventeen regions, age, and occupational status) was additionally evaluated with Holm’s sequential correction. Effect sizes used Cohen’s *d*, judged against the benchmarks of Cohen (1988) and Ferguson (2009). For the focal nonsignificant gender contrast on within-person scores, two one-sided equivalence tests (TOST; Lakens, 2017) assessed whether the observed effect was statistically smaller than a smallest effect size of interest of *d* = 0.20, the conventional small-effect benchmark (Cohen, 1988) and the upper bound of the design’s stated sensitivity (Section 3.1). To separate a genuine priority from individual differences in scale use, two within-person analyses were added: first, each respondent’s eighteen ratings were centered on that respondent’s own mean (ipsative scores), removing overall positivity and acquiescence, and the competency means and the gender comparison were recomputed on these scores; second, each respondent’s ratings were converted to within-person ranks (1 = most important), and mean ranks were compared across competencies and by gender. Analyses were conducted in Python (pandas, NumPy, SciPy, statsmodels), and because the study is exploratory, interpretation emphasizes effect sizes over binary significance.

### 3.4 Robustness checks

Three checks examined how robust the first-place standing was. First, the rating of crisis management was compared with those of the second- and third-ranked competencies using paired-samples *t*-tests and Wilcoxon signed-rank tests, with effect sizes expressed as the within-pair standardized mean difference (*d*_*z*) and the matched-pairs rank-biserial correlation. Because *d*_*z* standardizes by the within-pair standard deviation whereas the between-gender Cohen’s *d* standardizes by the pooled between-group standard deviation, the two effect sizes are not on the same metric and are not directly comparable. Second, a nonparametric bootstrap (10,000 resamples of respondents drawn with replacement) recomputed the eighteen item means in each resample to estimate the probability that crisis management attained the highest mean. The bootstrap followed standard resampling procedure (Efron and Tibshirani, 1994). Third, because all items were rated near the top of the scale, the compression of the ratings was quantified by the proportion of responses at the scale maximum and by item-level skewness.

## 4 Results

### 4.1 The competencies are closely spaced

All competencies were rated near the top of the scale, so the differences among them are small. The eighteen item means fell within a narrow band (5.446–6.056), 64.6% of all ratings were 6 or 7, and every item was left-skewed (mean skewness = −0.95). For crisis management, 41.6% of respondents selected the scale maximum. Such compression limits the size of any gap that can emerge between competencies, so the rankings below are best read as an ordering of closely spaced, uniformly high ratings rather than as evidence of large separations. We therefore treat the position of crisis management as a question of salience and stability rather than of dominance.

### 4.2 Crisis management ranks first among closely spaced competencies

Addressing RQ1, and read against the close spacing just described, crisis management received the highest mean importance rating among the eighteen competencies (*M* = 6.056, SD = 1.038; 95% CI [5.99, 6.12]; Table 1). It did not stand apart, however. Communication skills followed at *M* = 6.003 and social skills at *M* = 5.970, leaving a gap of only 0.053 between the first and second positions, and the 95% confidence interval for that gap included zero ([−0.013, 0.119]). Paired comparisons confirm that the lead is not statistically meaningful. Crisis management did not differ from communication skills (paired *t*(999) = 1.58, *p* = 0.115; Wilcoxon *Z* = −1.56, *p* = 0.120; *d*_*z* = 0.05), and although it exceeded social skills (paired *t*(999) = 2.64, *p* = 0.008, 95% CI of the difference [0.022, 0.150]; Wilcoxon *Z* = −2.87, *p* = 0.004), the difference was negligible (*d*_*z* = 0.08). Crisis management is thus the highest-rated of several closely spaced top competencies rather than a uniquely dominant one.

**Table 1:** Importance ranking of the eighteen competencies (*N* = 1,000).

Rank	Competency	*M*	SD
1	Crisis management	6.056	1.038
2	Communication skills	6.003	1.092
3	Social skills	5.970	1.087
4	Adaptability and flexibility	5.927	1.040
5	Cognitive skills	5.919	1.051
6	Self-awareness	5.890	1.096
7	Collaborative leadership	5.840	1.106
8	Knowledge	5.839	1.109
9	Transformational ability	5.827	1.115
10	Sustainability	5.818	1.093
11	Global leadership	5.780	1.163
12	Ability to handle complexity	5.683	1.093
13	Digital competence	5.669	1.131
14	Values	5.648	1.115
15	Financialization	5.531	1.119
16	Organizational skills	5.475	1.123
17	Customer-centric skills	5.459	1.109
18	Human orientation	5.446	1.189

*M*, mean importance; SD, standard deviation. Competencies are ordered by mean importance.

### 4.3 The ranking is broadly stable across subgroups

Addressing RQ2, the high ranking of crisis management held across the cohort (Table 2). It ranked first or second within most academic fields. The exceptions were medicine and pharmacy, arts and physical education, and education, where it placed third, fourth, and eighth; the eighth-place position rests on the smallest field cell (education, *n* = 22) and should be read with caution. It ranked first in both the capital area and elsewhere, and at or near the top in every age group and occupational category. The overall ordering of competencies was highly concordant across the eight fields (Kendall’s *W* = 0.82, *χ*^2^(17) = 111.8, *p* < 0.001), and overall importance did not differ by field (*F*(7, 729) = 1.358, *p* = 0.220; field analyses use the 737 respondents with a declared field), region (capital vs. non-capital *t*(998) = −0.55, *p* = 0.585; across-region *F*(16, 983) = 1.253, *p* = 0.221), or age (*F*(2, 997) = 0.204, *p* = 0.816). Occupational status showed a significant mean-level difference (*F*(4, 995) = 4.320, *p* = 0.002), the only test in this five-test family to remain significant under Holm’s sequential correction (Holm-adjusted *p* = 0.009; all other subgroup tests were nonsignificant before and after correction), but it did not alter the rank-stability pattern in Table 2, where crisis management remained at or near the top within every occupational category. The ranking therefore holds broadly across subgroups, even where mean levels differed. To check that this stability reflected a shared priority structure and not only similar mean levels, we compared the full eighteen-competency profile across the three age bands. The profiles were highly congruent (pairwise Pearson *r* = 0.92–0.93; Kendall’s *W* = 0.94, *χ*^2^(17) = 47.96, *p* < 0.001), with crisis management ranking first in each band. A two-way analysis of the ratings (age band × competency) showed a negligible interaction (partial *η*^2^ = 0.002; *F*(34, 17,946) = 0.91), indicating that the shape of the competency profile does not meaningfully vary by age band, so the departure from perfect profile correlation reflects noise rather than systematic developmental difference.

**Table 2:** Importance and rank of crisis management across subgroups.

Subgroup	*n*	Crisis-mgmt *M*	Rank (of 18)
Full sample	1,000	6.056	1
Humanities	151	6.000	2
Social sciences	143	6.259	1
Education	22	5.909	8
Natural sciences	78	5.936	2
Engineering	201	6.119	1
Medicine/pharmacy	56	5.804	3
Arts/physical Ed.	60	5.867	4
Other	26	6.077	1
Capital area	589	6.051	1
Non-capital	411	6.063	1
14–19	332	6.096	1
20–23	334	6.039	1
24–28	334	6.033	1
Students	263	6.072	1
Employed	239	5.958	1
Freelance	48	6.000	2
Not employed	210	5.890	4
Other	240	6.292	1
Men	500	5.932	1
Women	500	6.180	1

Rank = position of crisis management among the eighteen competencies within that subgroup. Subgroups with *n* ≥ 20 shown. Cells with very small *n* (e.g., education, *n* = 22) yield unstable ranks and should not be interpreted substantively.

### 4.4 Gender differences and a response-style test

Turning to gender (RQ3), apart from an overall level difference by occupational status (Section 4.3), gender produced the most systematic, competency-specific mean-level differences, and it raised rather than lowered the rating of crisis management. In raw scores, women rated crisis management higher than men (men *M* = 5.93, women *M* = 6.18; t(998) = −3.80, *p* < 0.001, *d* = −0.240; signs follow the convention of Table 3, in which negative values indicate higher ratings among women), and seven of the eighteen competencies were significantly higher among women under Bonferroni correction, concentrated in the social, communicative, adaptive, self-awareness, values-related, cognitive, and crisis-management domains (Table 3). These differences are small (|d| ≤ 0.36), and the pattern of women scoring higher across many competencies coincides with a higher overall use of the upper scale: women’s mean rating across all eighteen items was 0.19 points above men’s. To separate a different ordering of priorities from this difference in scale use, the gender comparison was recomputed on within-person (ipsative) scores. After removing each respondent’s overall positivity, most of the gender differences disappeared. Only social skills remained significantly higher among women (*d* = 0.25), and the gap in crisis management was no longer significant (*d* = 0.09, *p* = 0.166, 95% CI [−0.04, 0.21]). Because a nonsignificant test does not itself establish the absence of a difference, we added the equivalence test specified in Section 3.3: the two one-sided tests rejected effects of |d| ≥ 0.20 (TOST *p* = 0.038), and the 90% confidence interval of [−0.02, 0.19] fell entirely within the equivalence bounds. The adjusted gender gap in crisis management is therefore statistically smaller than a conventionally small effect, although true effects below that bound, up to roughly *d* = 0.19, cannot be excluded. The method therefore discriminates rather than flattens. It isolates one genuine difference, a relatively higher female priority on social skills consistent with social-role accounts (Eagly and Wood, 2012; Koenig et al., 2011; Paustian-Underdahl et al., 2014; Mohan et al., 2022), while indicating that the gender gap in crisis management is consistent with a difference in scale use rather than in priority, within the bounds established by the equivalence test. We favor the response-style reading of the crisis-management gap on two grounds: women’s elevation was systematic across nearly all competencies, the signature of scale use, not of a single differentiated priority; and the same correction left the relational emphasis on social skills intact, so it discriminates among differences rather than erasing them all.

**Table 3:** Gender comparison of the eighteen competencies (men *n* = 500, women *n* = 500).

Competency	Men *M* (SD)	Women *M* (SD)	*t*	*p*	*d*
Crisis management*	5.93 (1.11)	6.18 (0.95)	−3.80	0.0002	−0.240
Communication skills*	5.85 (1.16)	6.16 (1.00)	−4.59	0.0000	−0.290
Social skills*	5.78 (1.18)	6.16 (0.96)	−5.61	0.0000	−0.355
Adaptability and flexibility*	5.81 (1.17)	6.05 (0.88)	−3.70	0.0002	−0.234
Cognitive skills*	5.81 (1.14)	6.03 (0.94)	−3.36	0.0008	−0.212
Self-awareness*	5.78 (1.16)	6.00 (1.02)	−3.07	0.0022	−0.194
Collaborative leadership	5.74 (1.18)	5.94 (1.01)	−2.93	0.0035	−0.185
Knowledge	5.74 (1.17)	5.94 (1.03)	−2.83	0.0047	−0.179
Transformational ability	5.73 (1.19)	5.93 (1.03)	−2.82	0.0049	−0.178
Sustainability	5.72 (1.17)	5.92 (1.00)	−2.85	0.0045	−0.180
Global leadership	5.70 (1.23)	5.86 (1.09)	−2.23	0.0257	−0.141
Ability to handle complexity	5.65 (1.11)	5.72 (1.08)	−1.01	0.3113	−0.064
Digital competence	5.64 (1.21)	5.69 (1.05)	−0.70	0.4848	−0.044
Values*	5.51 (1.20)	5.79 (1.01)	−3.94	0.0001	−0.249
Financialization	5.50 (1.14)	5.57 (1.10)	−0.99	0.3229	−0.063
Organizational skills	5.45 (1.17)	5.50 (1.07)	−0.65	0.5174	−0.041
Customer-centric skills	5.41 (1.18)	5.51 (1.03)	−1.51	0.1307	−0.096
Human orientation	5.35 (1.26)	5.54 (1.11)	−2.45	0.0144	−0.155

*d*, Cohen’s *d*; negative values indicate higher ratings among women. *Significant after Bonferroni correction (*α* = 0.0028).

### 4.5 Robustness: resampling and within-person analyses

Resampling supported the first-place standing while bounding its certainty. Across 10,000 bootstrap resamples of respondents, crisis management produced the highest mean importance in 93.8% of resamples and ranked among the top two in 99.8%. Communication skills was the only other competency to reach the highest mean with appreciable frequency (6.0%).

Table 4 summarizes these robustness checks alongside the compression diagnostics introduced in Section 4.1.

**Table 4:** Robustness checks on the first-place standing of crisis management (*N* = 1,000).

Robustness check	Method	Result
Crisis management vs. communication skills (rank 2)	Paired *t*-test; Wilcoxon; *d*_*z*	*t* = 1.58, *p* = 0.115; Wilcoxon *p* = 0.120; *d*_*z* = 0.05
Crisis management vs. social skills (rank 3)	Paired *t*-test; Wilcoxon; *d*_*z*	*t* = 2.64, *p* = 0.008; Wilcoxon *p* = 0.004; *d*_*z* = 0.08
Probability crisis management has the highest mean	Bootstrap (10,000 resamples)	0.938 (top two 0.998)
Ratings of 6 or 7 on the seven-point scale	Proportion of responses	64.6% overall; 41.6% at scale maximum for crisis management

*d*_*z* = within-pair standardized mean difference; the matched-pairs rank-biserial correlations for the two paired comparisons were 0.08 and 0.14, respectively. Bootstrap resamples (10,000) were drawn from respondents with replacement.

Two within-person analyses test whether the ranking and the gender pattern depend on individual differences in scale use (Table 5). Centering each respondent’s ratings on their own mean (ipsative scores) left the competency ordering essentially unchanged. Crisis management remained highest (ipsative *M* = +0.29), ahead of communication (+0.24) and social skills (+0.20), which indicates that the ranking does not arise merely from some respondents using the top of the scale more than others. Within-person ranks placed crisis management first (mean rank 7.82 of 18), with only minor reorderings among the lower-ranked competencies. Even so, crisis management was the single most important competency for only about half of respondents (51.9% placed it at their personal maximum, often tied), a sign of how closely spaced the top competencies are. The broad gender differences reported in Section 4.4 did not survive ipsatization. Together with the resampling results, these analyses support a stable but closely spaced ordering.

**Table 5:** Within-person robustness: ipsative means and within-person ranks (*N* = 1,000), ordered by raw mean importance.

Competency	Raw M	Ipsative M	Within-person mean rank
Crisis management	6.056	+0.290	7.82
Communication skills	6.003	+0.237	8.07
Social skills	5.970	+0.204	8.36
Adaptability and flexibility	5.927	+0.161	8.77
Cognitive skills	5.919	+0.153	8.67
Self-awareness	5.890	+0.124	8.81
Collaborative leadership	5.840	+0.074	9.04
Knowledge	5.839	+0.073	9.05
Transformational ability	5.827	+0.061	9.22
Sustainability	5.818	+0.052	9.23
Global leadership	5.780	+0.014	9.26
Ability to handle complexity	5.683	−0.083	9.99
Digital competence	5.669	−0.097	10.02
Values	5.648	−0.118	10.24
Financialization	5.531	−0.235	10.95
Organizational skills	5.475	−0.291	11.10
Customer-centric skills	5.459	−0.307	11.26
Human orientation	5.446	−0.320	11.14

Ipsative scores center each respondent on their own eighteen-item mean. Within-person mean rank: 1 = most important within a respondent. Within-person mean ranks can reorder slightly relative to mean importance, particularly among the lowest-rated competencies, because of within-respondent ties. Bootstrap results are reported in Table 4.

## 5 Discussion

### 5.1 The salience of crisis management

Before interpreting these patterns, we restate that the single-wave design cannot separate cohort, age, and period effects. “Generation Z” here denotes the sampled cohort, and the findings describe its current priorities rather than a demonstrated generational effect. Among this cohort, ratings of the eighteen competencies were uniformly high and closely spaced, and within this compressed range crisis management received the highest mean importance, was closely followed by the next-ranked competencies, and ranked at or near the top across most subgroups, though its margin was small and not statistically distinguishable from the second-ranked competency. Korea’s social, economic, and geopolitical context provides one interpretive backdrop, not a tested explanation, for this standing. Korea’s risk environment (Section 2.3) keeps crisis-relevant exposure salient in national life, conditions under which crisis competence may be a broadly held leadership expectation. Because such exposure is society-wide, not confined to a particular discipline, region, or workplace, it offers one way of understanding why the priority appears broadly across subgroups. Korean organizational scholarship likewise describes leadership as embedded in affect-based informal ties (jeong) and group harmony (Yang, 2006), consistent with the high standing of communication and social skills alongside crisis management in this cohort. The reading complements, and does not replace, the generational-socialization account (Bundy et al., 2017). That the cohort rates highly a competency understood as a learnable, phase-spanning repertoire, not a fixed trait (Pearson and Clair, 1998; Wooten and James, 2008) is consistent with an expectation that leaders can be prepared for disruption.

### 5.2 Contextualizing crisis-management priorities

Chronic societal instability plausibly heightens the salience of crisis competence in this cohort’s leadership expectations, a reading that the breadth and stability of the priority support, that cross-national comparison can test, and that a single-country design cannot confirm (Hobfoll, 1989). We develop it cautiously. The data describe stated priorities and measure neither systemic risk nor any causal link to it, and prior governance scholarship that treats crisis response as a distributed public task (Boin et al., 2016; Eid et al., 2023) is not tested here. Because the reading is pitched at the societal rather than the individual level, within-cohort occupational variation does not test it: crisis management remained salient even among the not-employed, where it placed fourth (*M* = 5.89) rather than first, and we measure no individual-level resource variables that would let us model resource exposure directly. The mechanism can be adjudicated only by the cross-national comparison identified above.

Two things should be kept separate. The first is what the study demonstrates: a stable, compressed prioritization of crisis management across this cohort. The second is what it proposes for future testing, namely that chronic societal instability may contribute to that prioritization. The second is an interpretive conjecture, not a result; as Section 2.4 notes, a single-country design cannot adjudicate it. The decisive test of the contextual account is a cross-national comparison that includes a lower-risk cohort, in which the macro-environment account predicts a lower or less broadly shared standing for crisis management. Any policy reading is correspondingly tentative, hypothesis-generating rather than prescriptive.

### 5.3 Gender dynamics in leadership expectations

The gender result requires care. In raw ratings, women scored higher on relational, communicative, and adaptive competencies, a pattern that at first reading aligns with social role theory (Eagly and Wood, 2012). However, women also used the upper scale more overall, and once this difference in response style is removed through a within-person analysis, almost all of these gaps, including the gap in crisis management, are no longer significant, and for crisis management the adjusted gap was also statistically smaller than a conventionally small effect in an equivalence test (Section 4.4), though effects below *d* = 0.20 cannot be excluded. What survives is a specifically relational emphasis (social skills), which is consistent with social-role accounts. Within those bounds, the broader pattern is more consistent with a difference in how the scale was used than with a difference in the priorities themselves. Either way, the gender difference did not change the overall ranking of crisis management. An alternative reading cannot be ruled out by the present design: women may genuinely hold higher leadership expectations across the board, as a substantive response to structural inequality rather than a scale-use artifact, in which case the within-person correction would remove this genuine effect along with response-style variance. Cross-sectional, single-occasion data cannot adjudicate between the two accounts.

### 5.4 Theoretical and methodological implications

The central methodological lesson is visible in our own results. Had we reported only the raw ranking and the bootstrap, a reader would reasonably conclude that crisis management is this cohort’s top leadership priority; the paired comparison and the within-person correction show that such a conclusion overreads the data. The effect sizes underlying these contrasts are small (*d*_*z* ≤ 0.08 for the paired lead; |d| ≤ 0.36 for the raw gender gaps), so the interpretive weight they can bear is limited. A first-place mean in a compressed distribution marks salience, not dominance, and a raw gender gap can reflect scale use rather than priority. The discipline of testing whether a lead is statistically meaningful and whether a group difference survives a within-person correction is rarely applied in generational leadership research, and applying it changes what the same data are taken to show. The paper’s primary contribution is accordingly methodological: not that crisis management ranks first, but that ranking first, on its own, licenses far less than it is usually taken to.

These results also bear on how crisis competence is discussed. Prior accounts of the same framework placed crisis management at the periphery of millennial and Western leadership expectations (Ngayo Fotso, 2021, 2024a), so its first-place mean in this cohort is notable. The robustness analysis, however, cautions against reading that first-place mean as evidence of a distinct demand. Crisis management is salient, but within a narrow band it is not statistically separated from communication. For the study of leadership perception this matters: a competency can rise to the top of what a cohort expects of leaders without being singled out above the next-ranked qualities, so evidence of a defining generational leadership demand requires more than a first-place mean.

The study is among the first large-scale (*N* = 1,000) East Asian Generation Z studies to subject a single competency’s apparent priority to this combination of paired, bootstrap, and within-person tests. Applied readings follow only tentatively. Because these expectations are broadly shared across subgroups, not segmented by field or region, efforts that engage this cohort can treat crisis-related competence, communication, and adaptability as common ground. These implications extend beyond the firm: whether crisis competence is treated as a baseline expectation rather than an optional addition is equally a question for university curricula, public-sector selection, and youth-policy design. Perceived fit between a person’s own expectations and an organization’s profile is a well-established driver of attraction and commitment (Kristof-Brown et al., 2005). A more consequential reading is definitional, though the small margins involved keep it conditional. If crisis competence has shifted, for this cohort, from a specialized function reserved for executives to a baseline expectation of any leader, then development that treats it as an optional module aims at the wrong target: the capacity to contain disruption is expected to be built into ordinary leadership rather than added to it. Whether this reflects the movement of a single competency or a broader change in the leadership prototype is a question about latent structure that the present design, focused on salience rather than dimensionality, does not resolve. Because the top competencies are closely spaced and tightly compressed, these practical readings are tentative: the data indicate broadly shared, closely spaced expectations rather than a single competency valued decisively above communication or adaptability.

### 5.5 Limitations and future directions

Several limitations bear on these conclusions, and they differ in weight. Those that most directly bound our central claim are the compressed rating distribution and the single-item, single-occasion measurement; those that qualify scope rather than the core finding include the quota sample and the reach of the contextual interpretation. The ratings were strongly compressed toward the top of the scale: all eighteen means fell between 5.446 and 6.056 on a seven-point scale, and the gap between the first- and second-ranked competencies was only 0.053. This narrow spread compresses variance, so the stability of the ranking should be read as an observed pattern in compressed data, not as evidence of a large substantive gap between the top competencies. We report within-person (ipsative and rank-based) analyses to mitigate this, but did not field forced-choice or best-worst formats, which would recover more variance at the design stage. Each competency was measured with a single item, which limits reliability estimation (Bergkvist and Rossiter, 2007; Wanous et al., 1997) and assumes the abstract single-word labels (e.g., “financialization,” “human orientation,” “values”) were understood uniformly. Conclusions are accordingly restricted to stated importance ratings of labeled competencies rather than validated constructs, and we cannot verify uniform understanding among respondents as young as fourteen. Pooling respondents aged 14–28 also places secondary-school students and working adults in a single frame. Although crisis management ranked first in all three age bands (Section 4.3), developmental and life-stage heterogeneity within the cohort remains a limitation. The data are single-wave, cross-sectional, and self-reported, so cohort, age, and period effects are perfectly confounded and cannot be separated, and common-method bias cannot be ruled out (Podsakoff et al., 2003; Rudolph et al., 2018); the contextual interpretation offered above is therefore exploratory rather than directly tested, and the results should not be read as evidence of a generational effect. The sample was quota-based rather than probability-based. Item-order and fatigue effects also warrant note. Because this is a secondary analysis, we cannot confirm whether the eighteen items were presented in randomized order; but if the item columns reflect the fielded order, the highest-rated competency appeared last, the opposite of what a fatigue account predicts, and the correlation between item position and mean importance was weak (*r* = −0.19), so order effects are unlikely to explain the ranking.

Three measurement issues bound these conclusions more tightly than the single-item caveat alone conveys. First, single items are most defensible for concrete, narrowly bounded constructs (Bergkvist and Rossiter, 2007; Diamantopoulos et al., 2012); the focal construct, crisis management, is arguably more concrete, but the abstract lower-ranked labels are not, and because the provider’s pretest assessed general comprehension rather than age-graded content validity, we cannot verify uniform interpretation among respondents as young as fourteen; the lower positions in particular warrant caution. Second, and more fundamentally, rating scales do not force respondents to trade competencies off against one another, which can lift ratings across the board and generate the ceiling and compression we observe; the conclusion that crisis management is salient but not dominant may therefore partly reflect the rating format rather than the structure of preferences. Best-worst and forced-choice formats mitigate this and yield more reliable prioritization for a given number of responses (Kiritchenko and Mohammad, 2017; Mühlbacher et al., 2016). Our within-person rank analysis (Section 4.5) imposes a partial ordering in the same direction, but because those ranks are formed after respondents have already rated every competency highly, they are anchored on those ratings and approximate a post-hoc forced ranking rather than the design-stage trade-off that best-worst scaling imposes; they therefore cannot replace a design that forces trade-offs at measurement. Under such a design the prediction concerns rank stability rather than margin width, since best-worst formats widen margins by construction: if crisis management retains first place under best-worst scaling, the priority would survive a more demanding design; if the ranking reorders, the present ordering would be shown to depend on the rating format’s failure to enforce trade-offs. That comparison is the decisive design test of whether the ranking survives forced trade-offs.

The within-person (ipsative) correction carries its own costs. Centering each respondent on their own mean imposes a sum-to-zero constraint across competencies that can distort covariances and validity estimates (Brown and Maydeu-Olivares, 2013; Bürkner et al., 2019) and removes a person-level mean that may itself be substantively meaningful (Fischer and Milfont, 2010; Hanel et al., 2020). The correction may therefore under-detect genuine priority differences as well as remove response-style variance. For this reason we report raw scores, ipsative scores, within-person ranks, and bootstrap resampling together and read the gender result as convergence across these lenses rather than as an artifact of any one of them.

Future work can address these constraints directly. The cohort-age-period confound calls for multi-wave longitudinal or cross-sequential designs that re-survey the cohort and add younger and older comparison cohorts, separating durable generational effects from life-stage change. Beyond the within-person analyses reported here, the compression invites forced-choice or best-worst scaling at the design stage to compel prioritization and recover variance, alongside multi-item competency scales and scenario-based behavioral indicators in place of single items. The governance argument can be tested with multi-level designs that pair cohort attitudes with data from policy actors, central and local government, educational institutions, and employers, to examine whether and how shared crisis-competence expectations are met across the governance system. Cross-national replication would further separate the Korean macro-environment from broader generational effects.

## 6 Conclusion

Among eighteen twenty-first-century leadership competencies, Korean Generation Z rated all eighteen highly, and within a narrow, compressed range crisis management stood at the top of a closely spaced set of competencies. It remained at or near the top in most academic-field, regional, age-band, and occupational subgroup analyses, with lower placements in a few small field cells. Gender produced the most systematic raw differences across competencies, but a within-person analysis showed these to be largely a matter of overall scale use rather than relative priority. Because the margins among the top competencies were small and the gap to the second-ranked competency was not statistically significant, these results are best read descriptively: crisis management occupies a salient and stable position within this cohort’s leadership expectations, without being decisively separated from communication or social skills. This pattern can be interpreted cautiously against Korea’s broader risk environment, but the present design does not establish that this environment causes the observed preferences.

The study makes two contributions. The first is methodological. Claims that a generation rates some competency the top priority, and reports of raw gender differences, are common in this literature but are rarely tested for whether a lead is statistically meaningful or whether group differences survive a within-person correction. The present analyses show why both tests matter and point toward forced-choice and within-person designs as remedies. The second is substantive. In a large East Asian Generation Z sample, crisis management is robustly salient: it is the highest-rated of eighteen competencies, ranks at or near the top across most subgroup analyses, and holds first place with high stability under resampling, which is notable because prior accounts of the same framework placed it at the periphery of the leader profile. The finding advances the evidence base on what a younger, non-Western cohort expects of leaders, a question that matters for research on leadership perception and generational change. Its limits are equally clear, however. Crisis management is statistically tied with communication, so it is prominent without being a distinct priority, and its apparent elevation among women is consistent with a response-style interpretation rather than a difference in priority, though the present design cannot fully rule out a genuine difference. These readings are exploratory and bounded by the cross-sectional, single-item design, and they provide a baseline for longitudinal and cross-national work on young people’s leadership expectations.

## Data Availability

The datasets presented in this article are not readily available because they derive from a commercial research panel and include subgroup information on respondents, including minors, so public deposition is not possible. The fully anonymized dataset supporting the conclusions of this article is available from the corresponding author upon reasonable request. Requests to access the datasets should be directed to jhchoi@assist.ac.kr.
